# Knowledge Management Framework for Emerging Infectious Diseases Preparedness and Response: Design and Development of Public Health Document Ontology

**DOI:** 10.2196/resprot.7904

**Published:** 2017-10-11

**Authors:** Zhizun Zhang, Mila C Gonzalez, Stephen S Morse, Venkat Venkatasubramanian

**Affiliations:** ^1^ Fu Foundation School of Engineering and Applied Science Department of Chemical Engineering Columbia University New York, NY United States; ^2^ Mailman School of Public Health Department of Epidemiology Columbia University New York, NY United States; ^3^ Center for the Management of Systemic Risk Columbia University New York, NY United States

**Keywords:** EIDs, public health, systems engineering, knowledge representation, teleological function, knowledge management, ontology, semantic reasoning

## Abstract

**Background:**

There are increasing concerns about our preparedness and timely coordinated response across the globe to cope with emerging infectious diseases (EIDs). This poses practical challenges that require exploiting novel knowledge management approaches effectively.

**Objective:**

This work aims to develop an ontology-driven knowledge management framework that addresses the existing challenges in sharing and reusing public health knowledge.

**Methods:**

We propose a systems engineering-inspired ontology-driven knowledge management approach. It decomposes public health knowledge into concepts and relations and organizes the elements of knowledge based on the teleological functions. Both knowledge and semantic rules are stored in an ontology and retrieved to answer queries regarding EID preparedness and response.

**Results:**

A hybrid concept extraction was implemented in this work. The quality of the ontology was evaluated using the formal evaluation method Ontology Quality Evaluation Framework.

**Conclusions:**

Our approach is a potentially effective methodology for managing public health knowledge. Accuracy and comprehensiveness of the ontology can be improved as more knowledge is stored. In the future, a survey will be conducted to collect queries from public health practitioners. The reasoning capacity of the ontology will be evaluated using the queries and hypothetical outbreaks. We suggest the importance of developing a knowledge sharing standard like the Gene Ontology for the public health domain.

## Introduction

The 2014 Ebola epidemic in West Africa reminded the public health community again of the weaknesses in preparing for and responding to emerging infectious diseases (EIDs). The epidemic directly affected the health and economies of multiple countries in West Africa for 2 years and resulted in 11,299 deaths among 28,599 suspected infections [1]. The initial international response was regarded as slow and uncoordinated by many experts [2], an indication of the poor application of the lessons learned from prior global pandemics.

Effective coordination and communication of information among different stakeholders are necessary components of a strong response to an EID outbreak [3]. Public health coordination and communication requires not only sharing resources and specialties but also sharing, managing, and using knowledge effectively. This is a recognized challenge in practice [4-8]. Knowledge sharing and management is not a single government task. It needs the collaboration of multiple groups across several sectors. Such effort, however, is usually hindered by geographical, temporal, and political constraints. Lack of a strong public health infrastructure in many countries and the persistent problems in our global health governance structure could exacerbate the crisis and complicate the collaboration [4]. The spatial-temporal dynamics of outbreaks further complicate the real-time preparedness and response processes [9-11]. Moreover, how to use the knowledge from prior pandemics to make a prompt decision under current conditions perplexes the public health community.

Different approaches have been employed to address this challenge. Recent progress includes influenza information management [12], public health meta-knowledge analysis [13], and public health surveillance [14]. Semantic reasoning has been used to address the spatial-temporal difficulties of epidemic management [9]. However, advances in the knowledge management of public health have been limited. In this work, we demonstrate how to apply systems engineering concepts to develop a knowledge management framework facilitated by ontology and semantic reasoning.

The public health system is a complex adaptive system [6]. We can tackle its complexity using a systems engineering–based approach [15]. Systems engineering, first proposed by Bell Telephone Laboratories in the 1940s [16], describes an interdisciplinary engineering methodology that focuses on how to design and manage complex systems. It emphasizes the joint effect of system components, their dynamical interactions, and the environment. Systems engineering promotes the development of risk management in various industries, including aerospace, defense, chemical, and nuclear. Venkatasubramanian [17] discusses the necessity of the systems engineering idea for risk management in a complex system. Leveson [18] develops a systems engineering–based modeling framework to assess risks of engineered systems. There are other similar efforts in different domains [19-21]. EID preparedness and response resemble risk management in many engineering disciplines. Recently, systems engineering concepts have gained considerable attention in the public health community. The National Academy of Engineering and the Institute of Medicine have advocated the widespread application of systems engineering tools [22]. Systems engineering methods such as Markov models are used to enhance public health preparedness [23].

As a result, we propose a novel systems engineering–inspired, ontology-driven knowledge management approach. In this work, we demonstrate how to develop the ontology and semantic rules to manage knowledge and support decision making. This ontology could also serve as a part of other applications, such as a public health training or practice tool. Its flexibility enables the integration with other ontologies.

## Methods

### Overall Architecture

**Figure 1:**
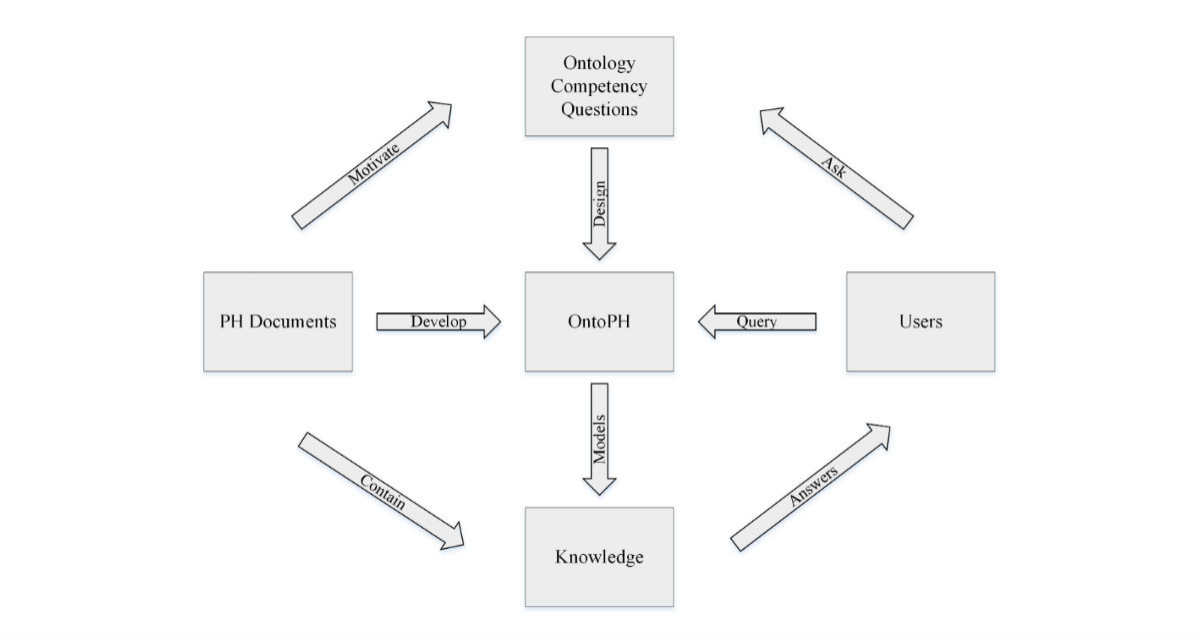
Systems engineering inspired ontology-driven knowledge management approach.

Public health knowledge management aims to systematically manage tasks and support decision making, which view implicit and explicit knowledge as key strategic resources [24]. Knowledge management needs storage, retrieval, and utilization of public health knowledge. We propose the ontology-driven knowledge management approach as shown in Figure 1, which decomposes public health documents to elements of knowledge and stores them in an ontology, namely, the Public Health Document Ontology (OntoPH). OntoPH was developed using ontology competency questions as guidance. Grüninger and Fox [25] state that an ontology should answer competency questions proposed based on the motivation of the ontology. Competency questions define the terminology and specify the definitions and constraints of the terminology. Knowledge is modeled using the terminology and retrieved via semantic rules. An inference engine accesses knowledge models and assembles and manipulates elements of knowledge in the ontology to draw conclusions about EID preparedness and response.

Public health knowledge is mainly preserved in public health documents, which include guidelines, procedures, and academic publications. They are the most important media to share, store, and manage knowledge because they are vetted, high-quality, generated by an authoritative content source, verifiable by a trusted source, and up to date and regularly updated [5]. In order to support decision making, OntoPH’s corpus should meet at least 2 requirements: breadth and depth. Breadth means the corpus should cover many, if not all, fields that are involved in public health decision making. Depth means the corpus should contain not only global-level guidelines but also local-level procedures.

### Function-Based Knowledge Representation

The first task is to represent knowledge preserved in public health documents. Effective knowledge storage and retrieval requires a knowledge representation, which addresses both hierarchical complexity and semantic heterogeneity. The hierarchical complexity of public health knowledge is rooted in the multiple layers of public health activities. Public health practitioners need different chunks of knowledge in various contexts to prepare for and respond to EIDs. Health workers in the clinic, for example, demand knowledge about disease diagnosis, whereas the department of health wants to know how to manage and coordinate. Knowledge always serves some purposes. The health workers’ knowledge leads to accurate diagnoses. The department of health’s knowledge achieves effective emergency response. Multiple layers of public health activities are linked via their purposes. For example, to better respond to emergencies, departments of health require the health workers to diagnose diseases more effectively.

Semantic heterogeneity, on the other hand, is the result of the cross-reference of public health knowledge, which is a mixture of various fields such as medical science, epidemiology, biology, and engineering [8]. For instance, the knowledge of physician training lies in the intersection of medical science (ie, what skills to train) and management science (ie, how to train). Nonetheless, the 2 aspects share the same purpose (ie, training physicians for better EID preparedness). A recent study by Venkatasubramanian and Zhang [26] finds that complex system activities usually have 4 common purposes: communication, decision making, processing, and sensing. Training, as part of education, is an important type of implementation activities.

One can resolve both hierarchical complexity and semantic heterogeneity by identifying the purpose of knowledge, for a piece of knowledge could serve different purposes under different conditions. Venkatasubramanian and Zhang [26] identify the importance of means-end relation in complex system risk management and propose a systems engineering framework to explicate the relation. Adopting this idea, our approach models elements of knowledge based on their means-end relations. We use teleological functions to represent the purposes of knowledge elements. Unlike mathematical functions that map a set of inputs onto a set of permissible outputs, teleological functions emphasize the means to realize a goal by indicating the common purpose between 2 connected entities. The 4 common purposes induce 4 types of teleological functions. A function-based knowledge representation has been used in many fields including engineering [27-30] and data science [31].

**Figure 2:**
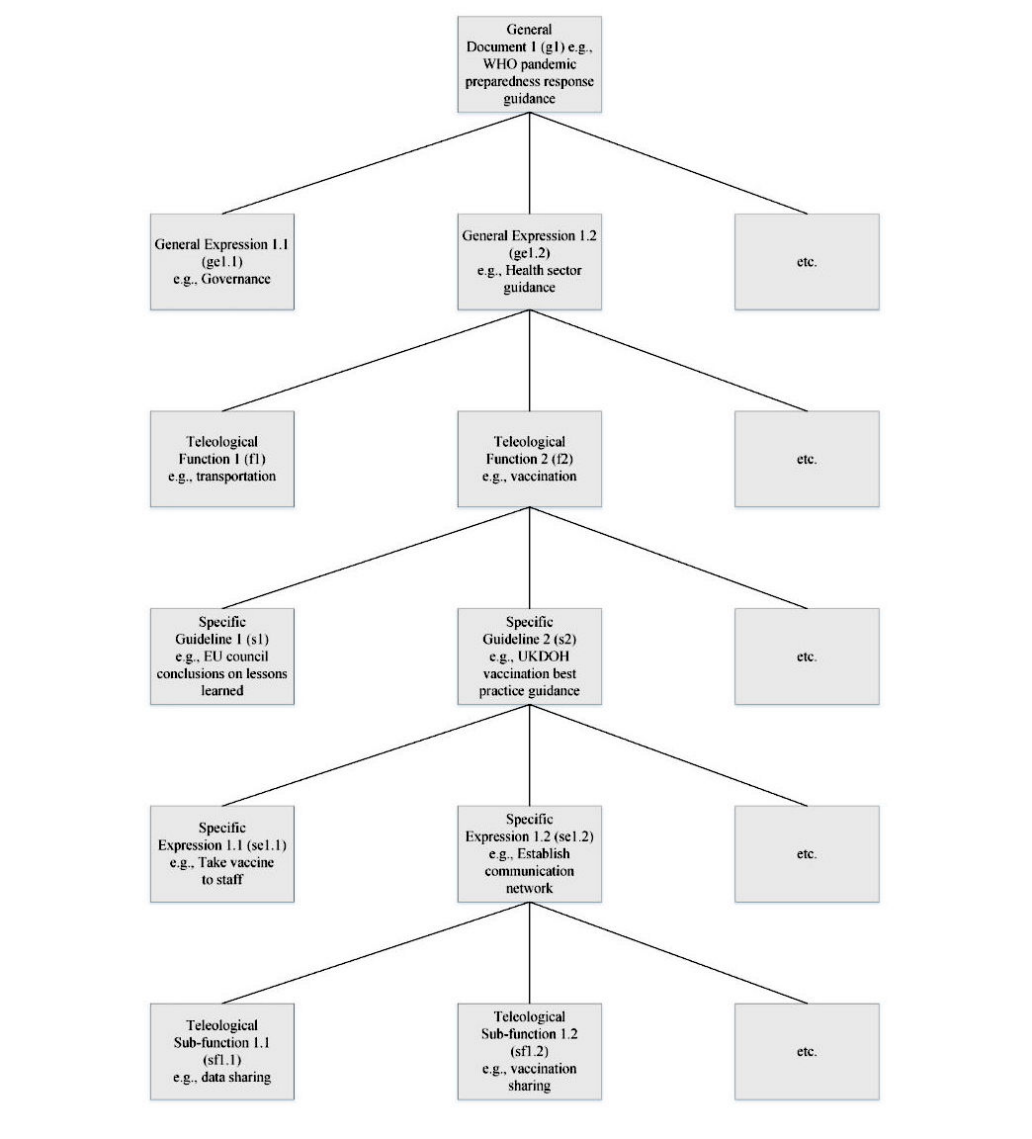
The tree structure of function-based knowledge.

To develop such a function-based knowledge representation, we first classify public health documents into 2 categories, general documents that contain general public health principles and specific documents that store evidence-based procedures. There exists a gap between the 2 types of documents: general documents are usually too general to implement, whereas specific documents are mostly event-specific thereby limiting their usefulness for new events. We organize knowledge of general documents as a teleological function of that of specific documents: *knowledge*_general doc_= *f(knowledge*_specific doc1_, *knowledge*_specific doc2_,...), where *f* is a teleological function. Specific activities expand a general guideline with specific recommendations. For example, since the 2009 influenza A H1N1 pandemic, many specific documents have discussed vaccination preparedness and distribution [32,33]. The World Health Organization (WHO) also has issued general guidelines for vaccination preparation during the pandemic [34]. The function vaccination describes activities related to vaccination preparedness and distribution. Therefore, the equation can be rewritten as *knowledge*_[34]_ = *vaccination* (*knowledge*_[32]_, *knowledge*_[33]_), meaning that WHO guidelines about vaccination can be expanded with specific activities and, hence, bridge the gap. The function-based knowledge representation is depicted as a tree structure shown in Figure 2. The root of the tree is a public health document, and the leaves are the event-based procedures. A general document (eg, g1) contains general knowledge expressions (eg, ge1.1 and ge1.2). A general knowledge expression specifies a teleological function. For instance, the WHO guideline [34] points out roles of the health and nonhealth sectors in vaccination sharing and distribution activities. We can label this knowledge expression with a function vaccination (eg, f2). Specific guidelines (eg, s2) elaborate the teleological functions and define many specific knowledge expressions (eg, se1.2). Specific knowledge expressions can further indicate subfunctions (eg, sf1.2), which include detailed procedures and instructions. Unlike specific procedures, teleological functions are event independent. The same functions can apply to different events with similar fundamental lessons. The tree structure demonstrates how general documents and specific documents are linked via teleological functions. The function-based knowledge representation handles the hierarchical complexity through the tree structure of documents and manages the semantic heterogeneity by grouping distinct activities under the same function. Teleological functions define the scope and intention of the specific documents. They let a specific document elaborate a general document by adding actionable items.

### Ontology Development

#### Background

**Figure 3:**
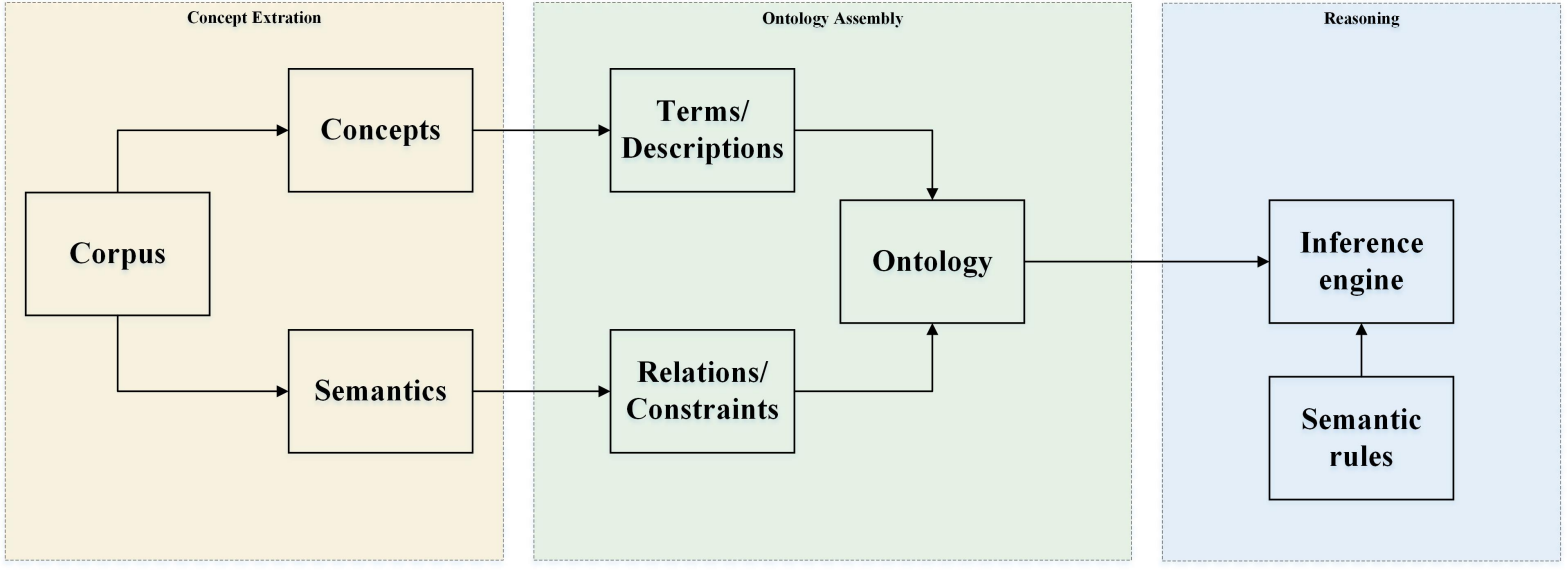
Ontology development process.

An ontology is a formal description of entities and their properties, relationships, and constraints [25]. It is widely used for the information system and knowledge management. An ontology consists of classes, individuals, and properties. Classes are a collection of concepts in the domain of discourse. Individuals are instances of each class. Properties are relations between classes, values restrictions, or instance descriptions in the domain of discourse. An ontology models knowledge by axiomatizing concepts as well as the relationships between them [35]. Knowledge is defined and organized in a layer style (see Multimedia Appendix 1). Terms with similar meanings are classified as synonyms. A list of synonyms is defined as a concept. Concepts form a hierarchy and are connected by relations. Concepts and relations constitute general axioms that represent the knowledge of discourse. Figure 3 shows the ontology development process, which consists of 3 steps: (1) concept extraction: extracting knowledge from the corpus; (2) ontology assembly: decomposing knowledge into terms, relations, constraints, and descriptions, integrating these components to form an ontology; and (3) reasoning: creating semantic rules to enable knowledge retrieval.

#### Concept Extraction

There are 2 concept extraction methods available: manual annotation and natural language processing (NLP) annotation. Manual annotation requires domain experts to review and annotate every term in the corpus per predefined criteria. Manual annotation provides high accuracy but requires tremendous human effort. On the other hand, NLP annotation automatically recognizes and classifies terms into predefined categories [36]. NLP annotation is much more efficient than manual annotation but at the cost of accuracy. Usually, an NLP-based information retrieval performs clustering or classification to identify key concepts. The performance is usually measured by precision or recall [37].

#### Ontology Assembly

OntoPH includes 199 classes, 78 properties, and 1234 axioms (see Multimedia Appendices 2-8). We developed the general structure of OntoPH based on the Legal Knowledge Interchange Format (LKIF) core ontology. The LKIF core ontology was developed by the European Project for Standardized Transparent Representations to extend a legal accessibility consortium to cater to a continuing need for a standard vocabulary of basic legal terms [38]. We expanded this legal term vocabulary to include public health vocabulary.

OntoPH is structured in a modularized nature. Modularization improves the reusability, scalability, and maintenance of an ontology [39,40]. OntoPH has 7 modules: space-time, agent, action, role, process, document, and event. Inheriting all modules, OntoPH core ontology has 9 main classes (Textbox 1). The space class defines spatial concepts such as region and nation. The time class describes temporal concepts such as time point or period. The resource class specifies resources used for public health preparation and response. The action class defines potential actions for an EID event. Actions are categorized regarding the 4 basic teleological functions: communication, control, implementation, and monitoring [26]. Subclasses of the action class represent specific functions under the 4 basic functions. The process class describes both continuous and discrete event flows. The agent class lists all the intelligent and nonintelligent agents involved in a process or an action. The description class describes the state and role of any agent, action, or process. The medium class summarizes different types of public health documents, such as legal or nonbinding documents. Last, the expression class represents the knowledge expressions of the documents.

**Ontology main classes and subclasses.**

Action

- Communication
- Control
- Implementation
- Monitoring

Agent

- Animal
- Human
- Organization
- Other agent
- Pathogen

Description

- Attribute
- Role

Expression

- Argument
- Assertion
- Assumption
- Declaration
- Evaluative proposition
- Evidence
- Fact
- Feedback
- Intention
- Knowledge
- Observation
- Qualification

Medium

- Document
- Sample

Process

- Continuous process
- Discrete process

Resource

- Equipment material
- Financial
- Human resource
- Intellectual tool

Space

- Area
- Space point

Time

- Period
- Time point

**Figure 4:**
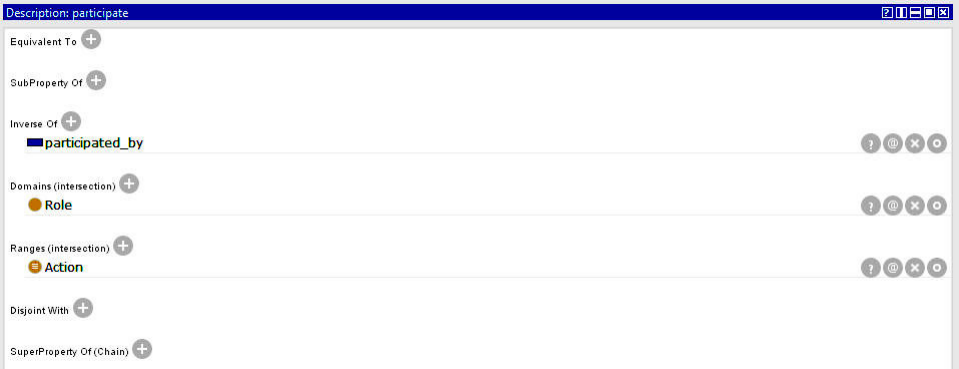
Protégé screenshot for property participate.

**Figure 5:**
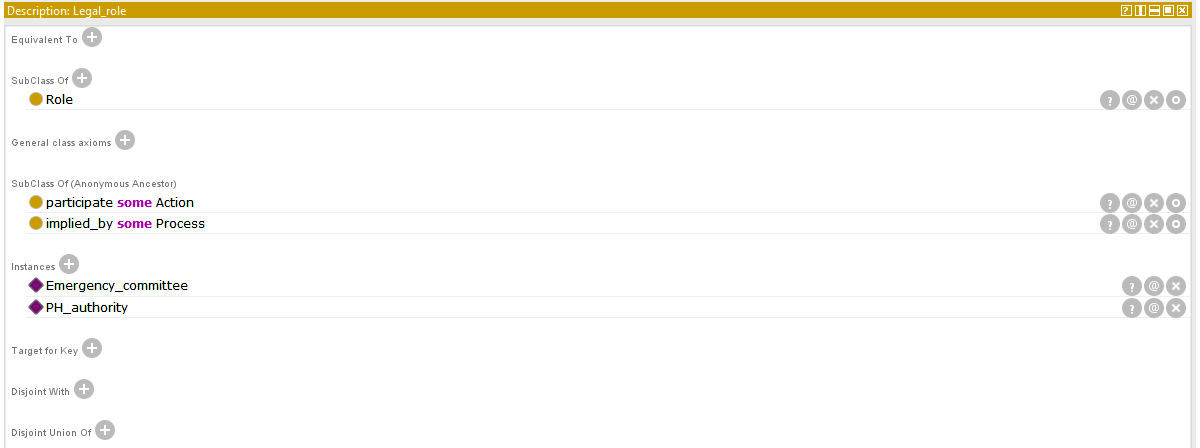
Protégé screenshot for individuals of Legal_role.

OntoPH properties (see Multimedia Appendices 6 and 7) define the relationships between classes and subclasses. For instance, participate (Figure 4) has a domain of role and a range of action, indicating that a role participates in some actions. This property has an inverse of participate_by. OntoPH contains individuals extracted from public health documents. For example, legal_role, a subclass of role, has individuals of emergency committee and public health authority (Figure 5).

#### Semantic Rules and Reasoning

OntoPH is developed using web ontology language (OWL) under the Protégé environment [41]. Logic-based semantic rules allow OWL to “exploit the considerable existing body of logical reasoning to fulfill important logical requirements” [42]. They imply answers to the competency questions. OntoPH answers 3 types of questions: (1) the relation between actions and roles, (2) the relation between roles and the condition of interest, and (3) the relation between actions and the condition of interest. OntoPH uses time, space, resource, and process classes to describe the conditions of an EID outbreak. Hence, we can construct the following informal competency questions:

1. What action must a role perform?
2. What are the roles specified by an action?
3. What are the actions required under a condition of interest?
4. What are the roles specified under a condition of interest?

Informal competency questions should be translated to a formal format so that an ontology can retrieve the elements of knowledge to answer them [25]. We denote *T*_ontology_ as a set of axioms in the ontology, *G*_ground_ as a set of ground instances, and *Q* as a first-order sentence using only predicates in the language of *T*_ontology_. We can formulate the formal translations for the 4 informal competency questions.

**Figure 6:**
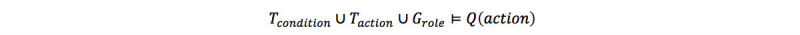
The formal expression of competency question 1.

**Figure 7:**
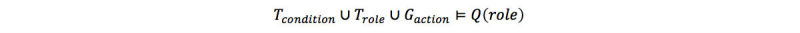
The formal expression of competency question 2.

**Figure 8:**
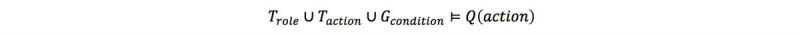
The formal expression of competency question 3.

**Figure 9:**
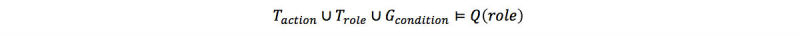
The formal expression of competency question 4.

1. Let *Q(action)* denote a sentence that describes some actions. Given a ground formula *G*_role_ defining instances of a role, determine the possible actions, as shown in Figure 6.
2. Let *Q(role)* denote a sentence that describes some roles. Given a ground formula *G*_action_ defining instances of an action, determine the possible roles, as shown in Figure 7.
3. Let *Q(action)* denote a sentence that describes some actions. Given a ground formula *G*_condition_ defining instances of a condition, determine the possible actions, as shown in Figure 8.
4. Let *Q(role)* denote a sentence that describes some roles. Given a ground formula *G*_condition_ defining instances of a condition, determine the possible roles, as shown in Figure 9.

Semantic rules link axioms *T* with instances *G* and entail a first-order sentence *Q*, which is the answer to the competency question.

Semantic rules are created using semantic web rule language (SWRL), a rule language for the semantic web. SWRL rules apply unary predicates for describing classes and data types, binary predicates for properties, and some special built-in n-ary predicates [43]. An example SWRL rule is shown in Textbox 2.

**A simple example of semantic web rule language rule.**

(Person(?x), hasParent(?x,?y), hasParent(?x,?z), hasSpouse(?y,?z) → childOfMarriedParents(?x)

**Figure 10:**
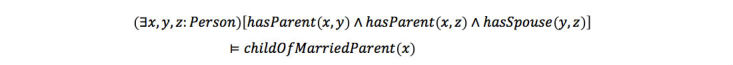
The formal expression of someone is a child of married parents.

This rule describes the assertion that someone is a child of married parents. Letters with a question mark (eg, *?x*) denote variables. Person(*?x*) indicates that a variable *x* is a person. The binary relation hasParent(*?x*, *?y*) indicates that person *x* has a parent *y*. The formal formula is shown in Figure 10, which reads: there exists persons *x*, *y*, and *z*. If *x* has parent *y*, and *x* has parent *z*, and *y* and *z* are spouses, then *x* is a child of married parents. SWRL rules translate natural language assertions into computable forms (Figure 10).

**Figure 11:**
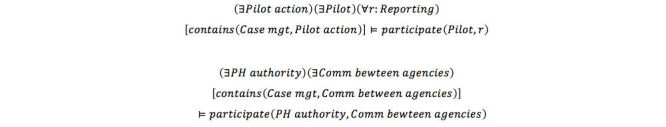
The formal expression of pilot in command action.

We create SWRL rules in 3 steps. First, public health experts review documents and identify knowledge expressions. For example, the WHO Technical Advice for Case Management of Influenza A (H1N1) in Air Transport (WHO Advice Air Transport) [44] is a WHO-issued guideline for air transportation case management. It specifies the procedures that the pilot in command should follow when a suspicious case is identified. We identify a knowledge expression pilot_in_command_action under the expression class. Second, public health experts create logic expressions for knowledge expressions. This intermediate step translates a procedure into a formal representation. For example, the pilot_in_command_action can be written as logic expressions, as shown in Figure 11.

Logic expressions and natural language are interchangeable. The first expression in Figure 11 shows that WHO Advice Air Transport contains specifications about pilot actions. The pilot in command should report any suspicious activities on the flight. The second expression in Figure 11 shows that WHO Advice Air Transport requires communication between agencies. The public health authority should communicate with other agencies. Third, public health experts work with ontology engineers to develop the SWRL rules based on the logic expressions from step 2. Textbox 3 shows the SWRL rule created for the same example. The rule first states the knowledge expression and its parent document. Then, it specifies the roles (Pilot and PH_authority) and the expected actions.

**Semantic web rule language rule for the pilot_in_command_action example.**

Guideline(Case_management_H1N1_Airtransport_guidance), Knowledge(Pilot_in_command_actions) → contains(Case_management_H1N1_AirTransport_guidance, Pilot_in_command_actions)

Nonhealth_sector(Pilot), Reporting(?reporting), contains(Case_management_H1N1_AirTransport_guidance, Pilot_in_command_actions) → participate(Pilot, ?reporting)

Legal_role(PH_authority), Interactive_network(Communication_between_agencies), contains(Case_management_H1N1_AirTransport_guidance, Pilot_in_command_actions) → participate(PH_authority, Communication_between_agencies)

**Figure 12:**
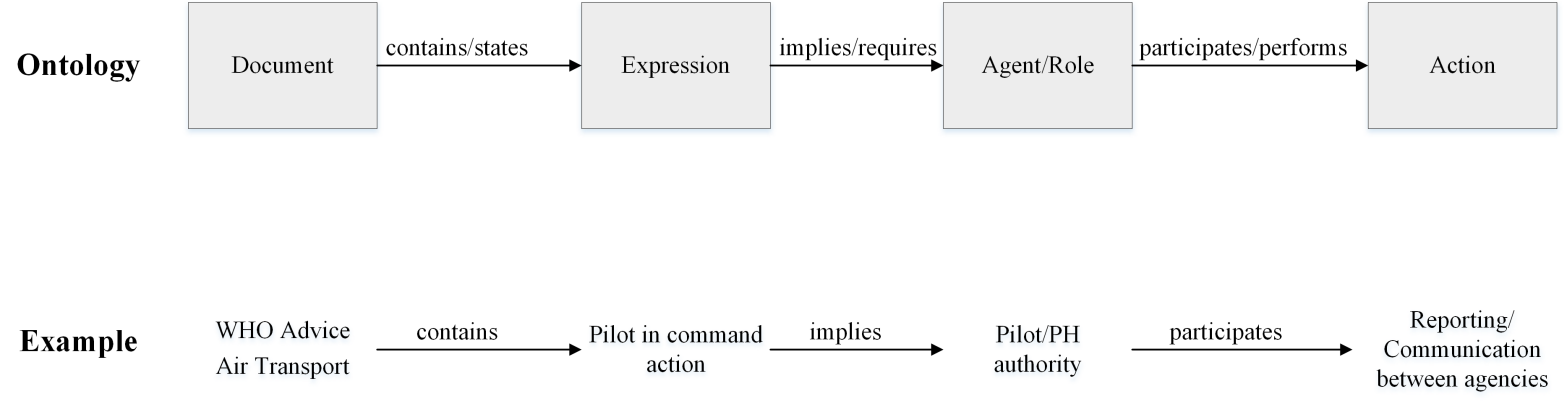
An inference process.

Logical inference connects documents with knowledge expressions. An inference process is depicted in Figure 12. WHO Advice Air Transport carries many knowledge expressions. One of them informs the chief pilot’s actions for an EID emergency during a flight mission. This piece of knowledge then implies that pilots and public health authorities should report suspicious cases and communicate with each other in time.

**Figure 13:**
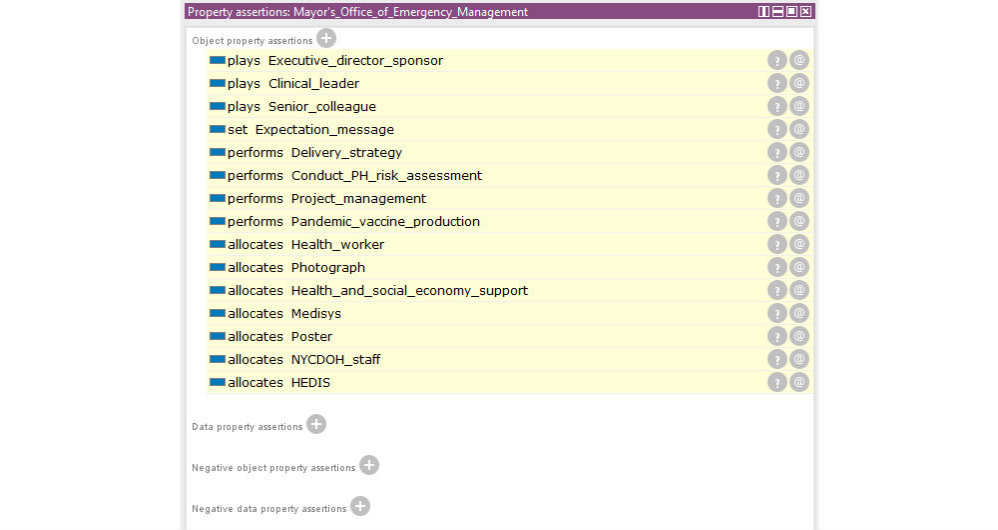
Reasoning results for Office_of_Emergency_Management.

Reasoning results are presented per individual. Figure 13 shows the reasoning results of Mayor’s Office of Emergency Management under the department class. Given an individual, we obtain a list of sentences, such as “Mayor’s Office of Emergency Management performs delivery strategy.” These sentences in fact are the elements of knowledge.

## Results

### Concept Extraction Results

The corpus, with 135,946 words in total, consists of the US Code [45], federal level regulations [32,33,46,47], international health regulations [34,44,48,49], and pandemic evaluations of outbreak responses [50,51]. They cover all types of public health documents aforementioned. The US Code is the generic legal document, which ensures that the ontology aligns with laws. The federal regulations and the international health regulations are guidelines regarding surveillance, transportation, and preparedness. The evaluations are chosen per disease. H1N1 and West Nile Virus are 2 specific diseases chosen for illustration. These 2 cases were selected because they are well-studied recent emerging diseases with an impact on health resources both locally and globally. In addition, their impacts on health and geographical coverage are both significant. We wanted to evaluate case examples where the primary infection risk is associated with different infection transmission routes in order to evaluate the potential for having a unified framework for EIDs.

We implemented a hybrid concept extraction approach. NLP methods are used to preprocess the corpus. By removing stop words and tagging the parts of speech, one can extract meaningful and most frequent terms and relations using text mining tools like KHCoder [52]. The classification work is done manually with 2 domain experts reviewing every term and relation and deciding their descriptions and constraints. OntoPH is built upon these terms and relations. Domain experts and ontology engineers work collaboratively to select and annotate documents. Such a team-based method has been used extensively in many scientific studies and applications, such as hazard and operability analysis in chemical engineering [53]. Such a team should be as small as possible while maintaining sufficient expertise. In a series of meetings, team members work together to select documents. Conflicts must be resolved before the list of documents is finalized. Each domain expert annotates a part of the corpus and reviews others’ annotations. This practice, therefore, keeps the corpus and annotation as objective as possible.

### Ontology Evaluation

The quality of ontology is critical. It affects not only the quality of reasoning results but also the effectiveness of the application. Ontology can be evaluated on many aspects, namely, vocabulary, syntax, structure, semantics, representation, and context [24]. Extensive research has been conducted to formally evaluate the quality of ontologies [24,54-57]. Among these methods, we follow the Ontology Quality Evaluation Framework (OQuaRE) approach [55], which adapts the International Organization for Standardization standards for Software Quality Requirements and Evaluation. OQuaRE assesses 6 characteristics and 39 subcharacteristics of an ontology using quality metrics. Quality metrics are composed of primitive and derived measurements. Primitive measurements are metrics that can be measured directly on the ontology, such as number of classes, number of relations, etc. Derived measurements are combinations of some primitive ones [55]. With a scale of 1 to 5 (1=not acceptable and 5=exceeds the requirement), it rates every aspect of an ontology. The final score is the arithmetic average of individual scores of all characteristics. The details of this method can be found in Duque-Ramos et al [55]. We include 30 out of the 39 subcharacteristics in our evaluation. The other 9 subcharacteristics, which require expert subjective assessment, are excluded. The evaluation results of the OntoPH core ontology are presented in Table 1. The evaluation indicates that the OntoPH core ontology is satisfactory, with an average score of 4. Problems have been found on redundancy and controlled vocabulary, mainly due to the relatively small corpus size.

**Table 1:** Ontology evaluation results.

Characteristics	Subcharacteristics	OQuaRE score
**Structural**		
	Formalization	5
	Formal relations support	4
	Redundancy	2
	Consistency	5
	Tangledness	4
	Cycles	5
	Cohesion	4
	Domain coverage	4
**Functional adequacy**		
	Controlled vocabulary	2
	Schema and value reconciliation	4.67
	Consistent search and query	4
	Knowledge acquisition representation	3.67
	Clustering	2
	Similarity	4
	Indexing and linking	4.5
	Results representation	5
	Text analysis	5
	Guidance	5
	Decision trees	4.5
	Knowledge reuse	4.28
	Inference	4.67
**Compatibility**		
	Replaceability	3.5
**Transferability**		
	Adaptability	3.5
**Operability**		
	Learnability	4.17
**Maintainability**		
	Modularity	3
	Reusability	4
	Analyzability	3.8
	Changeability	4
	Modification stability	4.2
	Testability	3.8

## Discussion

### Principal Findings

The possibility of using ontology and semantic reasoning in public health decision making has been recognized in literature [58]. In this work, we adapt this idea and our previous experience in knowledge management in the pharmaceutical industry [59] to derive a detailed methodology on how to develop such a tool. We introduce the systems engineering–inspired ontology-driven framework for public health knowledge management. We demonstrate how complex and heterogeneous public health knowledge can be modeled and stored in an ontology. Previous work has focused on local activities, such as activities within a health care network [60]. OntoPH extends the scope from local level to global/national level by focusing on general documents.

OntoPH’s strength is threefold. First, it stores public health documents knowledge as classes, relations, and instances. Public health documents, including guidelines, procedures, and academic publications, are important sources of knowledge. Even though medical records, geographic information system data, and disease information have been studied and stored in ontologies [60,61], to our knowledge, there is no ontology for public health documents. OntoPH provides this missing piece of public health knowledge management. Second, we present a flexible knowledge management framework. OntoPH implements a modularized structure, which ensures its extensibility. For example, the space-time module can be extended using time ontologies [60,62] and World Wide Web Consortium spatial ontologies [63]. It is also possible to add new modules. If disease information is needed, we can create a new disease module, which inherits the disease ontology [61]. This modularized structure makes OntoPH a potential generic public health knowledge center. Third, OntoPH can manage the hierarchical complexity and heterogeneity of public health knowledge. Elements of knowledge are effectively organized by the teleological functions that highlight the means-end relations.

This framework is most useful in low- and middle-income countries (LMICs). Lack of resources and public health experts in LMICs usually makes a knowledge management system difficult to implement. Nonetheless, OntoPH’s general knowledge is widely applicable. By expanding the data sources to include LMIC-specific knowledge [64] and connecting with other ontologies [61-63,65], OntoPH would become a useful tool to help LMICs respond to an outbreak quickly, both at the national and local levels.

Potentially, OntoPH can support decision making by answering user queries. For example, given an outbreak scenario, a user could list questions regarding disease identification, transmission prevention, disease control, and risk mitigation. With enough prestored knowledge, OntoPH could answer the list of questions by producing logical assertions with respect to each question.

### Limitations

At this stage, however, there are some limitations. First, the training document corpus is relatively small. Only 5 general documents and 7 specific documents are prestored due to the manual annotation constraint. It will require a concerted effort to annotate and develop a more extensive public health knowledge base for widespread application. Nonetheless, the current corpus is comprehensive enough for proof of concept. Second, the selection of documents is subjective. When the corpus size is small, the accuracy of reasoning results is dependent on the document selection rather than the knowledge base. Increasing the size of the corpus and precise query statement will improve reasoning accuracy in general. In addition, rule-based reasoning has its intrinsic limitations—semantic rules are subjective. SWRL rules rarely allow ternary relations, and that limits the power of the SWRL representation. Third, the current framework is restricted to public health documents, which lack information from various data sources, such as geographic information system data, news articles, social media feeds, etc. This limits OntoPH’s real-time usage. Moreover, current knowledge representation would not be able to capture knowledge in research articles that do not fit in the knowledge model. However, the basic and domain ontologies, such as space-time, resource, role, and agent modules, contain fundamental public health knowledge, therefore, making the knowledge framework extendable to cover research articles. This, of course, requires further study of new knowledge representation. Potentially, a research article knowledge expression module could be developed and incorporated into OntoPH.

### Future Work

Future work will address the limitations and evaluate OntoPH’s reasoning capacity. Adopting artificial intelligence techniques would significantly reduce the human effort, and, thus, get rid of many of the limitations. Specifically, a term extraction module implementing NLP techniques such as topic modeling would enable automated concept classification of public health documents, reducing the amount of work required for annotation. Enriching data sources will improve OntoPH’s ability for real-time response. We plan to expand the corpus incorporating expert opinions. A survey for eliciting expert feedback on what to include in the corpus will be conducted. A systematic literature review on effectiveness of policy and interventions could help us determine what documents to include.

To evaluate this method, we will collect a list of general queries regarding general EID preparedness and response from public health experts and practitioners. Moreover, we will test OntoPH’s reasoning capacity on hypothetical outbreaks. These full-scale case studies will provide us with valuable information on how to improve the usage and accuracy of OntoPH decision support.

### Conclusion

In recent decades, many EID outbreaks and epidemics have resulted in considerable human disability and mortality, in part due to ineffective coordination or slow response at the start of the outbreak. Responding to EID outbreaks is intrinsically challenging due to the uncertainties associated with EIDs, specifically level of risk and potential for impact of its spread in a population. During an outbreak, evidence-based public health policies developed by public health authorities, legislators, and other government officials facilitate the implementation of a strong public health response. However, there are structural and political forces that prevent decision makers from making evidence-based policies in response to outbreaks. Therefore, it is necessary to have in place a mechanism to easily identify evidence in order to evaluate the consequences of public health or policy actions recommended to address these public health emergencies. An ontology framework for public health outbreak response will cut the time spent aggregating expert opinions during the initial stages of an outbreak. It would also assist public health administrators and government officials on next steps based on individual- and systems-level factors associated with the outbreak.

Our approach is a potentially effective methodology for EID preparedness and response. It manages complex knowledge via a function-based knowledge representation. It introduces a systematic way of storing, retrieving, and using public health knowledge. Accuracy and comprehensiveness of the ontology can be improved as more knowledge is stored. We advocate the public health community work toward the goal of developing a Gene Ontology-like [66] knowledge sharing standard. OntoPH demonstrates the possibility of knowledge management for EID emergency preparedness and response.

## Appendix

Multimedia Appendix 1 Ontology layer representation (Adapted from Cimiano [35]).

Multimedia Appendix 2 Ontology classes part 1.

Multimedia Appendix 3 Ontology classes part 2.

Multimedia Appendix 4 Ontology classes part 3.

Multimedia Appendix 5 Ontology classes part 4.

Multimedia Appendix 6 Ontology properties part 1.

Multimedia Appendix 7 Ontology properties part 2.

Multimedia Appendix 8 Ontology individuals (selected).

## Abbreviations

EID: emerging infectious disease
LKIF: Legal Knowledge Interchange Format
OntoPH: Public Health Document Ontology
OQuaRE: Ontology Quality Evaluation Framework
OWL: web ontology language
NLP: natural language processing
SWRL: semantic web rule language
WHO: World Health Organization
LMIC: low- and middle-income country
