# Supplementary figures and images for: Knowledge Management Framework for Emerging Infectious Diseases Preparedness and Response: Design and Development of Public Health Document Ontology
Source: JMIR Res Protoc. 2017 Oct 11;6(10):e196. doi: 10.2196/resprot.7904 (PMC5656775; doi:10.2196/resprot.7904)

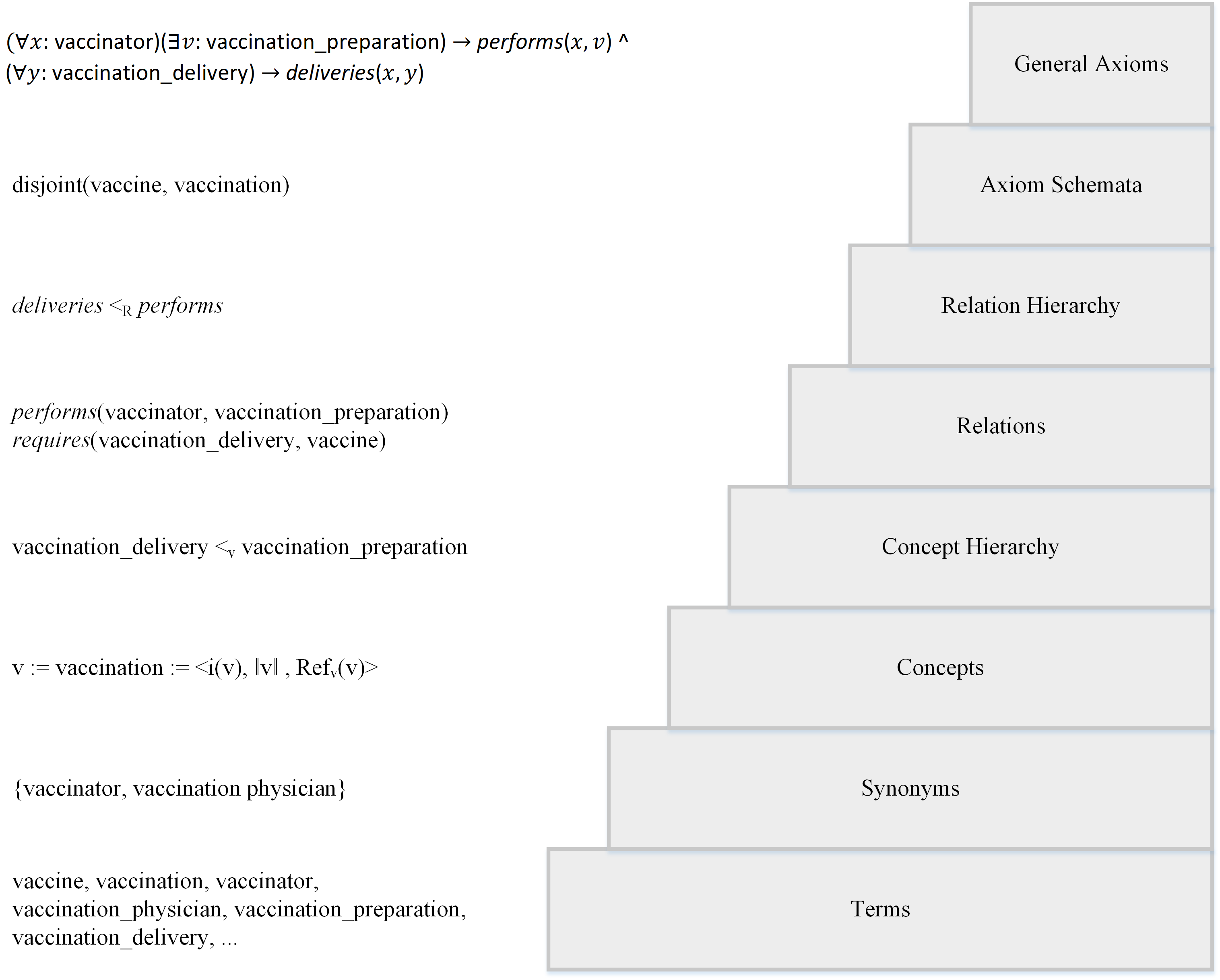

Supplement: Multimedia Appendix 1 [file resprot_v6i10e196_app1.png]

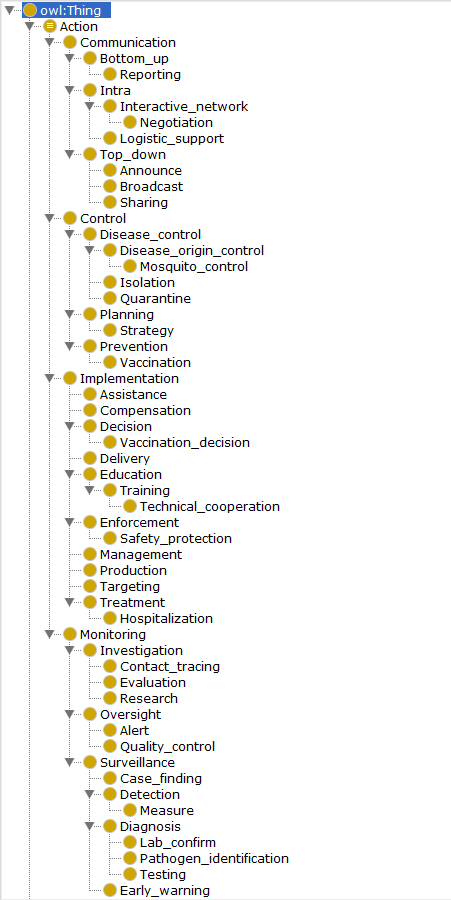

Supplement: Multimedia Appendix 2 [file resprot_v6i10e196_app2.png]

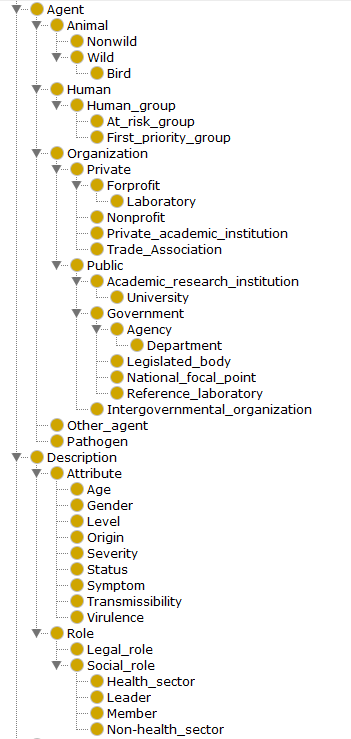

Supplement: Multimedia Appendix 3 [file resprot_v6i10e196_app3.png]

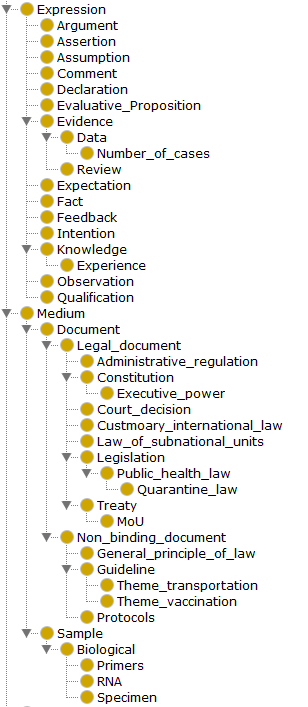

Supplement: Multimedia Appendix 4 [file resprot_v6i10e196_app4.png]

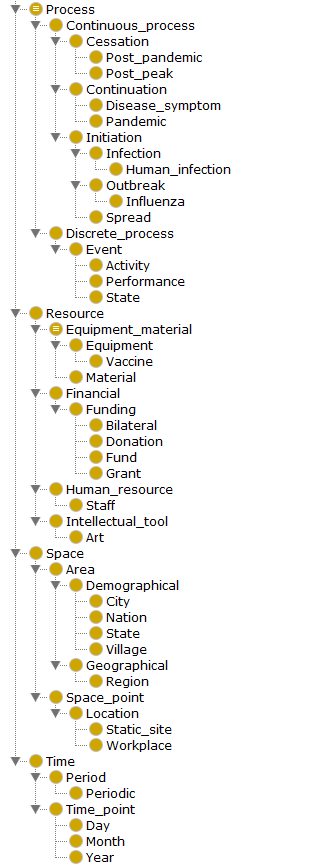

Supplement: Multimedia Appendix 5 [file resprot_v6i10e196_app5.png]

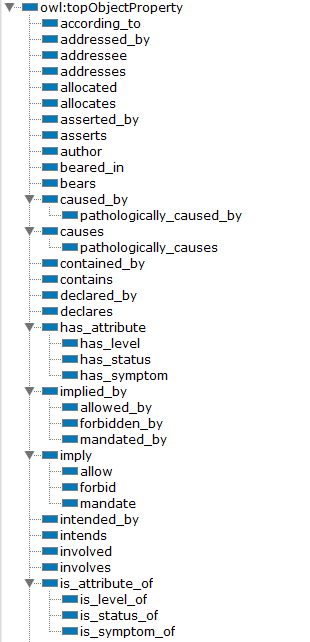

Supplement: Multimedia Appendix 6 [file resprot_v6i10e196_app6.png]

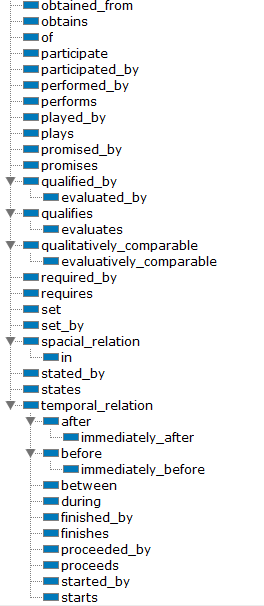

Supplement: Multimedia Appendix 7 [file resprot_v6i10e196_app7.png]

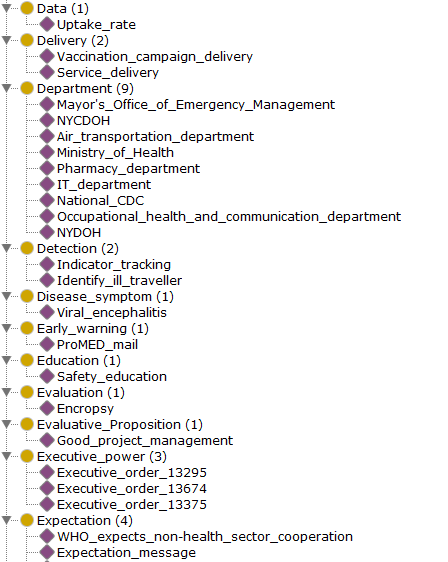

Supplement: Multimedia Appendix 8 [file resprot_v6i10e196_app8.png]
